# Targeting nuclear acid-mediated immunity in cancer immune checkpoint inhibitor therapies

**DOI:** 10.1038/s41392-020-00347-9

**Published:** 2020-11-20

**Authors:** Miaoqin Chen, Shiman Hu, Yiling Li, Ting Ting Jiang, Hongchuan Jin, Lifeng Feng

**Affiliations:** 1 Laboratory of Cancer Biology, Key lab of Biotherapy in Zhejiang Province, Cancer Institute of Zhejiang University, Sir Run Run Shaw hospital, School of Medicine, Zhejiang University, Hangzhou, Zhejiang 310016 China; 2 Department of Radiation Oncology, Sir Run Run Shaw Hospital, Medical School of Zhejiang University, Hangzhou, 310016 China

**Keywords:** Tumour immunology, Cancer therapy

## Abstract

Cancer immunotherapy especially immune checkpoint inhibition has achieved unprecedented successes in cancer treatment. However, there are many patients who failed to benefit from these therapies, highlighting the need for new combinations to increase the clinical efficacy of immune checkpoint inhibitors. In this review, we summarized the latest discoveries on the combination of nucleic acid-sensing immunity and immune checkpoint inhibitors in cancer immunotherapy. Given the critical role of nuclear acid-mediated immunity in maintaining the activation of T cell function, it seems that harnessing the nuclear acid-mediated immunity opens up new strategies to enhance the effect of immune checkpoint inhibitors for tumor control.

## Introduction

Cancer immunotherapy, exploiting to activate the immune system to fight malignancies, has become a major strategy for a long time. Early in 1891, Willam Cooley attempted to treat sarcoma via intratumoral injections of *Streptococcus pyogenes* and *Serratia marcescens* to activate immunity.^1^ Currently, the most commonly used strategy is the inhibition of immune checkpoints, particularly targeting the programmed cell death receptor-1 (PD-1), and its ligand, programmed cell death ligand-1 (PD-L1) and cytotoxic T lymphocyte-associated protein 4 (CTLA4).^2^ Nivolumab, pembrolizumab and camrelizumab are approved PD-1 blocking agents, while atezolizumab, avelumab, and durvalumab are approved PD-L1 blocking antibodies. Widely, these agents have been applied to the treatment of a variety of advanced tumors, including metastatic melanoma, non-small-cell lung cancer, gastric carcinoma, hepatocellular carcinomas, renal cell carcinoma, etc.^3–7^

Despite the dramatic therapeutic responses, these agents provide durable clinical responses only in nearly 20% of cancer patients as monotherapy.^8^ And there are many complicated mechanisms of primary or acquired resistance to immune checkpoint blockade. As the central of immune checkpoint inhibitors (ICIs) is to reactivate effector T cells, the resistance mainly resulted from inadequate infiltration or impaired function of T cells in the tumor microenvironment.^9^

As the first line of defense, innate immune responses are critical in the onset and maintenance of T cell responses, including T-cell-centered tumor immunity.^10^ Briefly, antigen-presenting cells (APC) uptake and present tumor antigens via major histocompatibility complex I (MHC-I), and thereby induce tumor-specific CD8^+^ T cell expansion. In addition, some APCs such as cDC1s (a subset of conventional dendritic cells) can also produce chemokines including CXCL9 and CXCL10 to recruit T cells to tumor microenvironment.^11^ Nature killer cells (NK cells), another important class of innate immune cells, can kill targeted tumor cells, secrete anti-tumor cytokines, and also contribute to the infiltration of cDC1s into tumors.^12^

The critical signals to induce immune responses include pathogen-derived toxins like LPS and ectopic genetic materials such as exogenous or cytosolic nuclear acids. Meanwhile, the so-called pattern recognition receptors (PRRs) are proteins which engaged in detecting the pathogen-associated molecular patterns and transduction the signalings of infectious agents elimination. More importantly, the organism activates the innate immune system relying on the PRRs mediated signaling.^13^ Nucleic acid sensors, which can detect extracellular or intracellular DNA or RNA as damage-associated molecular pattern signals, are the essential part of the PRRs. Upon activation, Nucleic acid sensors can induce type I interferons (IFNs). Type I IFNs are known for their critical roles in antiviral immune responses. The secreted Type I IFNs will act on producing and neighboring cells via IFNα/βreceptor 1 (IFNAR1, particularly high affinity for IFNβ) or IFNAR1-IFNAR2 heterodimer.^14^ Type I IFNs support cytotoxic T lymphocytes (CTLs) via several mechanisms. Firstly, they promote stimulating the maturation of DCs to improve cross-priming with T cells; secondly, they increase the expression of perforin 1 and granzyme B to boost functions of effect T cells; thirdly, they can inactivate the suppressive function of regulatory T (Treg) cells; Finally, Type I IFNs can prevent NK cells to eliminate antigen-activated CD8+ CTLs, and induce macrophages to release pro-inflammatory cytokines (such as IL-1β, and IL-18).^15^

As combinatorial strategies are required and enhanced, the combination of releasing the multiple brakes on T cells via ICIs and unleashing the innate immunity to activate T cell functions is a promising strategy to control tumor development. Here, we review the roles of nucleic acid sensors in innate or adaptive immunity, and the mechanisms which can influence the sensitiveness and effectiveness of the ICIs to anti-tumor. Our discussion focuses on the latest discoveries on the synergy of nucleic acid sensors-mediated innate immunity and ICIs as the promising signal in cancer immunotherapy.

## Nucleic acid sensors

### Retinoic acid-inducible gene-I (RIG-I) like receptors

**Fig. 1:**
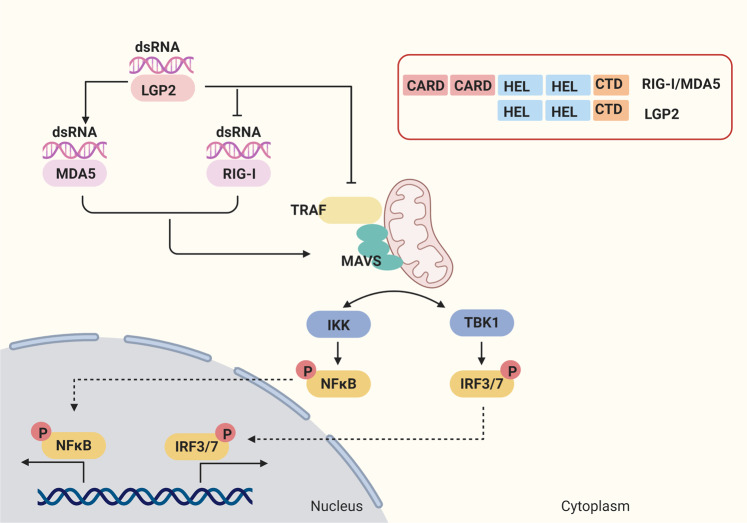
RIG-I-like receptors (RLRs) mediated signal transduction pathway. RIG-I-like receptors (RLRs) have been identified as important cytoplasmic RNA sensors, including RIG-I, MDA5, and LGP2. RLRs are common in sharing the DExD/H-box helicase domain and C-terminal domain (CTD), and RIG-I and MDA5 contain two N-terminal tandem caspase activation and recruitment domains (CARD), while LGP2 lacks CARD. RIG-I detects cytoplasmic viral short dsRNA that contains a 5′-triphosphate or 5′-diphosphate moiety, whereas MDA5 recognized long dsRNA structures. LGP2 can bind RNA ligands of RIG-I, interfering with IKK recruitment to MAVS through protein interaction or binding to RIG-I through a repressor domain directly, to inhibit the activation of RIG-I. LGP2 can increase the ability of MDA5 to form stable filaments on dsRNA to promote the MDA5- mediated pathway. LGP2 interacts with TRAF and disrupt its activity, which results in the disruption of IRFs and NFκB activation. Activated RIG-I and MDA5 induce the recruitment and polymerization of the adapter MAVS on the mitochondrial membrane. Then MAVS activates TBK1 as well as the IKK complex, which activates IRF3 and IRF7, and NF-κB. And the gene expression of IFNs, pro-inflammatory cytokines and chemokines is induced to defend viral and modulate the immunity

RIG-I like receptors (RLRs), including RIG-I, melanoma differentiation- associated protein 5 (MDA5), and laboratory of genetics and physiology 2 (LGP2), are expressed in the cytosol of most immune and non-immune cells, including cancer cells.^16^ RLRs are common in sharing the DExD/H-box helicase domain and C-terminal domain(CTD), which are essential for the recognition of exogenous nuclear acids like viral RNAs. In addition, RIG-I and MDA5 contain two N-terminal tandem caspase activation and recruitment domains (CARD), which are responsible for recruiting in the downstream signaling components and resulting in the activation of immunity. However, LGP2 lacks CARDs, which leads to the functions of LGP2 that differ from RIG-I and MAD5 (Fig. 1).^17^

RIG-I was initially recognized as an antiviral sensor.^18^ In contrast to synthetic siRNAs, exogenous polyinosine-polycytidylic acid (poly I:C, the synthetic RNA) was shown to activate RIG-I to induce innate antiviral responses including IFNs production. RIG-I recognizes RNA in a sequence-dependent manner, with the dsRNA mainly end with 5′ triphosphate (5′ppp).^19^ Despite MDA5 shares high sequence similarity with RIG-I, it senses distinct groups of viral RNAs in a length-dependent manner. Several biochemical and structural studies revealed that the longer dsRNA has a stronger affinity to MDA5, which could keep more stable during nucleation kinetics of MDA5, and provide a platform for MDA5 to interact with downstream signaling molecules.^20,21^

Following the recognition of dsRNAs via CTD of RLRs, the structural rearrangements allow the CARDs to induce the recruitment and polymerization of the adapter mitochondrial antiviral signaling (MAVS) on the mitochondrial membrane, which serves as a scaffold for the activation of TNF receptor-associated factor (TRAF) family (TRAF2,3,5,6).^22^ Besides, the TRAF propagates the signal sequentially from MAVS to TANK-binding kinase 1 (TBK1) and IκB kinase (IKK), and activates the transcription factors interferon regulatory factor 3 (IRF3) and nuclear factor-κB (NFκB) to stimulate the transcription of type I IFNs and antimicrobial inflammatory cytokines, respectively (Fig. 1).^17^ Another RLRs member LGP2 was originally regarded as an inhibitor of RIG-I sensing IFN signaling via binding RNA ligands of RIG-I, interfering with IKK recruitment to MAVS through protein interaction or binding to RIG-I through a repressor domain directly.^17,23–25^ However, LGP2 null mice display reduced responses to several RNA viruses detected by MDA5.^24^ The high basal ATP hydrolysis activation of LGP2 enhances its RNA recognition capacity and diversity, which increases the ability of MDA5 to form stable filaments on dsRNA, and synergizes with MDA5 to increase the antiviral response.^26^ LGP2 could interact with TRAF and disrupt its activity, which results in the disruption of IRF3 and NFκB activation.^27^ Therefore, the function of LGP2 might be context-dependent.

RLRs expressed in non-immune cells including some cancer cells, and RLRs stimulation via their ligands mimic the viral infection could induce apoptosis through upregulation of the pro-apoptotic gene TRAIL and downregulation of the anti-apoptotic genes BCL2, BIRC3, and PRKCE via IRF3 and IRF7.^28,29^ Meanwhile, RLRs activation could stimulate innate and adaptive immune responses. On one side, RLRs ligands could mimic viral infection to activate dendritic cells (DCs) directly. On the other side, the activation of RIG-I in cancer cells could induce the production of proinflammatory cytokines such as CXCL10, IL-6, IFNβand the upregulation of the MHC-I expression in cancer cells, which could stimulate DCs and subsequently activate cytotoxic T cells.^30–33^ In addition, activated RIG-I could recruit the inflammasome adapter ASC, which activates caspase-1. Activated caspase-1 could induce the maturation of IL-1β, IL-18, and Gasdermin-D. Activated Gasdermin-D translocates to the plasma membrane and oligomerizes, which forms pores and initiates hypotonic cellular swelling and lysis.^34,35^

### Toll-like receptors

**Fig. 2:**
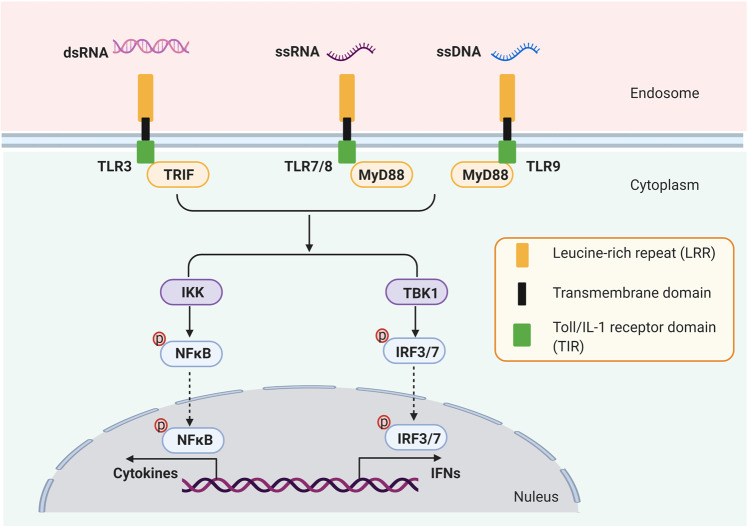
Toll-like receptors (TLRs) mediated signal transduction pathway. Toll-like receptors (TLRs) are comprised of at least 11 members. Among 11 members, TLR3, TLR7, TLR8, TLR9 were found to localize on endosome membrane and play an essential role in nucleic acid-sensing. TLR3 can detect dsRNA, and TLR7 and TLR8 recognized ssRNA. And unmethylated CpG motif of ssDNA can activate TLR9. TLRs are comprised of the N-terminal extracellular domain (ECD), transmembrane domain (TM), and C-terminal cytoplasmic Toll/IL-1 receptor domain (TIR). ECD domain is responsible for binding pathogen-associated molecular patterns (PAMPs), and the signaling cascades are conducted through the TIR domain, which recruits TRIF or Myd88 as adapters, to activate NFκB and IRFs

Another type of PRRs is Toll-like receptors (TLRs), comprising at least 11 members, playing important roles in inducing antimicrobial innate and adaptive immune responses.^36,37^ Among these members, TLR3, TLR7, TLR8, TLR9 were found to play an essential role in nucleic acid-sensing (Fig. 2).^36^ Generally, TLRs are comprised of the N-terminal extracellular domain (ECD), transmembrane domain (TM), and C-terminal cytoplasmic Toll/IL-1 receptor domain(TIR). ECD domain is responsible for binding pathogen-associated molecular patterns (PAMPs), and the signaling cascades are conducted through the TIR domain, which recruits TIR-domain-containing adapter protein including IFN-β (TRIF) or Myd88 as adapters, to activate NFκB and IRFs.^38^ For example, TLR3 is expressed on both cell and endosomal membranes in most innate immune cells. After recognizing virus-derived dsRNA^39,40^ and incomplete stem structures containing single-strand RNA ssRNA within endosomes,^41^ TLR3 oligomerizes and recruits the TICAM-1, and subsequently activates IRF3 and NFκB, to enhance the production of type I IFNs such as IFNβand proinflammatory cytokines such as TNF-αand IL-6.^42^ TLR7 and TLR8 are both endosome localized TLRs. They recognize viral ssRNA, imidazoquinoline compounds, and guanosine analogs, and recruit MyD88 to activate NFκB and IRF7.^43,44^ TLR7 is activated by synthetic small interfering RNAs (siRNAs),^45^ exosomal FMR1-AS1 lncRNA, ssRNAs such as microRNA let-7,^46^ ssRNAs derived from dead cells,^47^ and guanosine as well as its derivatives.^48^ In contrast, TLR8 senses the uridine, oligonucleotides, and the degradation products of ssRNAs.^49^ Despite the high similarity, TLR7 is expressed in plasmacytoid DCs (pDCs) and B cells while TLR8 mainly expressed in macrophages, monocytes, and cDCs.^41^ Activation of TLR7/8 via intratumoral agonist in a model of melanoma induced CCL2 production to facilitate the infiltration of M1 macrophages, CD8+ T cells, B cells.^50^

TLR9 is expressed dominantly in pDCs and B cells, and the unmethylated CpG motif of ssDNA is indispensable for TLR9 stimulation.^51^ By processing double strands DNA (dsDNA, such as chromosomal and mitochondrial DNA) into short ssDNA, DNases are essential to activate TLR9.^52^ Upon infection, TLR9 is critical to initiate innate immune responses via increasing proinflammatory cytokines and activating the cytotoxic NK cells and CD8+ T cells.^53^

### cGAS-STING pathway

**Fig. 3:**
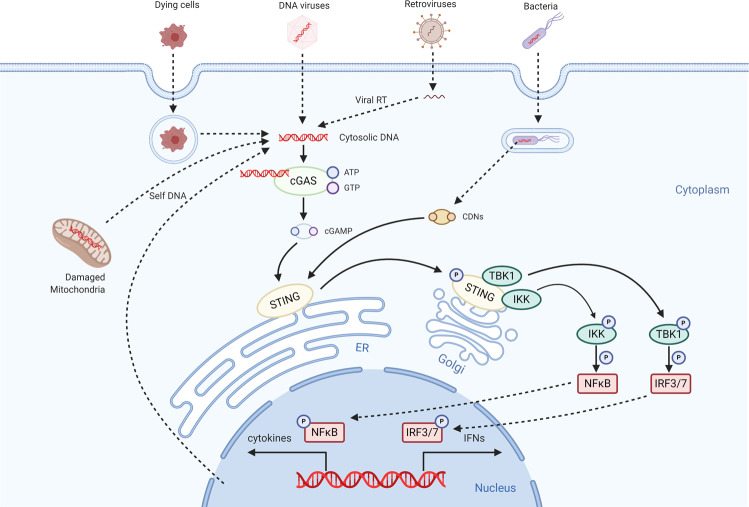
cGAS/STING- mediated signal transduction pathway. As the cytosolic DNA sensors, cGAS recognizes dsDNA from DNA viruses or dsDNA created by retroviruses. Following the detecting of DNA, cGAS synthesizes the second messenger cGAMP, which then binds to and activates STING on the endoplasmic reticulum (ER). In addition, STNG can sense CDNs. Activated STING translocates from ER to Golgi to form a complex with TBK1 or IKK. The activation of TBK1 or IKK induces the expression of type I IFN genes and other pro-inflammatory cytokines through the TBK1-IRF3/7 axis and NF-κB

Cyclic GMP-AMP synthase (cGAS) was first identified as a cytosolic double strands DNA (dsDNA) sensor in 2013.^54^ After binding to DNA directly, cGAS could covert GTP and ATP into cyclic GMP-AMP (cGAMP) to form the second messenger 2′3’-cGAMP for the activation of stimulator of interferon genes (STING).^55^ Once bound by 2’3’-cGAMP, STING translocates from ER to Golgi to form a complex with TBK1 or IKK. STING-activated TBK1 is able to phosphorylate IRF3, promoting IRF3 dimerization and translocation to the nucleus where it induces the transcription of many inflammation genes especially IFNβ. On the other hand, STING-activated IKK could phosphorylate IκBα, which leads to the translocation of NFκB to the nucleus, and then activates the transcription of proinflammatory cytokines (Fig. 3).^56^

Although foreign or self DNA can activate cGAS in a length-dependent manner,^57^ healthy cells can digest the host dsDNA precisely via DNase or compartmentalize the DNA in the nucleus or mitochondria to prevent the aberrant activation of cGAS-STING pathway.^56^ Respectively, mutation of DNase or STING, self-DNA accumulation caused by DNA damage, chromosome instability, mitochondrial dysfunction, and so on, could activate the cGAS-STING pathway aberrantly, thereby leading to various inflammatory diseases, including tumorigenesis.

### Inflammasomes

**Fig. 4:**
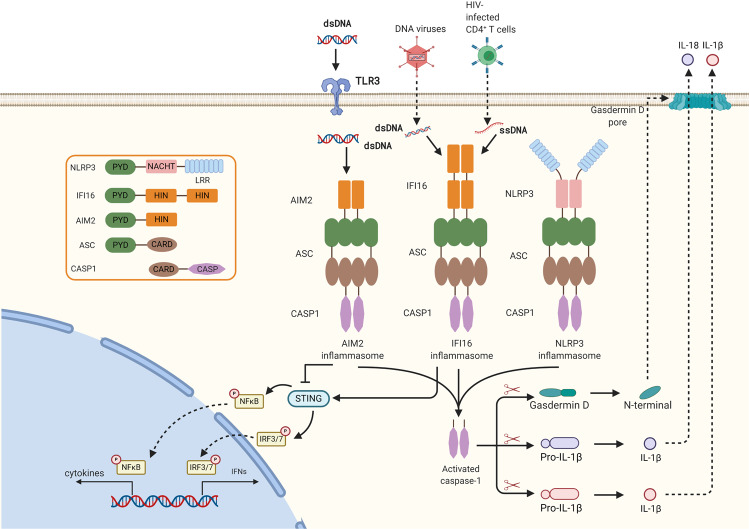
Inflammasome-mediated signal transduction pathway. The DNA sensor AIM2 and IFI16 is composed of an N terminal pyrin domain and C terminal HIN-200 domain. Cytosolic DNA induces activation of AIM2 or IFI16. The HIN-200 domain interacts with DNA, while the pyrin domain binds to the pyrin domain of ASC. CARD of ASC binds the CARD of pro-caspase-1, which activates caspase-1. Activated caspase-1 drives cleavage of pro-IL-1β, pro-IL-18, and Gasdermin D. In addition, activated IFI16 can recruit STING to activate IRF3 and NFκB to induce type I interferon

Inflammasomes are large multi-protein complexes to mediate the cytokines maturation and secretion, and pyroptosis. There are three components in a typical inflammasome: sensor, adapter, and effector. And the inflammasome sensors are usually divided into three classes: (1) Absent in melanoma 2 pyrin and hemopoietic expression, interferon-inducibility, nuclear localization, and characteristic 200 amino-acid domain (HIN200) containing protein (PYHIN) family, (2) nucleotide binding domain and leucine-rich repeats (NLR) containing protein family, (3) TRIM family member, Pyrin. Inflammasome sensors can detect the presence of pathogen-derived molecular, including nucleic acids, metabolites, and so on. Adapter proteins deliver the signal from the sensors to effectors through protein-protein interactions.^58^ And the effector drives the cleavage of pro-inflammatory cytokines (pro-IL-1β and pro-IL-18) and Gasdermin D, which lead to the secretion of mature interleukin-1β (IL-1β) and IL-18 and pyroptosis (Fig. 4).^59^ IL-18 has been shown to induce IFNγproduction, increase NK cell activity and T cell proliferation.^60,61^ IL-1 can promote Th17 polarization to secrete IL-17 alongside with IFNγ.^62^ Moreover, IL-1 can promote T cell proliferation, and suppress regulatory T cells and IL-10 production by T cells. Paracrine of IL-1 can contribute to mature DCs, which promotes CD8+ T cell responses.^63^

Among the three classes of sensors, PYHIN family members absent in melanoma 2 (AIM2) and interferon-γinducible protein 16 (IFI16) were regarded as DNA sensors.^64^ AIM2 consists of an N-terminal pyrin domain (PYD) and a C-terminal HIN-200 domain. dsDNA binds to the HIN domain of AIM2 to free the PYD domain of AIM2. And the free PYD domain interacts with the PYD-CARD-containing inflammasome adapter protein apoptosis-associated speck-like protein containing a carboxy-terminal CARD (ASC) into helical filaments, which leads the activation of the effector caspase-1.^65^ IFI16 containing a PYD and two HIN domains, is the first PYD-containing protein sensing DNA to mediate IFNβ induction.^66^ IFI16 recognizes dsDNA from invaded DNA viruses, ssDNA from HIV infected CD4+ T cells, and the nuclear damaged DNA from etoposide-treated keratinocytes.^67–69^ Apart from triggering ASC-caspase 1 dependent inflammasome to produce IL-1β,^69^ activated IFI16 can recruit STING to activate IRF3 and NFκB to induce type I IFN.^68^ Another study found that STING can interact directly with IFI16 and facilitate IFI16 ubiquitination on the lysine3/4/6 and degradation via the ubiquitin-proteasome pathway by recruiting the ubiquitin E3 ligase TRIM21.^70^ In addition, during *Mycobacterial* infection, activated AIM2 mediated inflammasomes can interact with STING and block the activation of TBK1 and IRF3, thus inhibiting the induction of IFN-β.^71^

The NLR containing protein 3 (NLRP3) has been well characterized to play important roles in viral infection and viral nucleic acid sensing.^64^ However, it is unclear whether NLRP3 directly senses viral DNA or RNA.

### Other nuclei acid sensors

Z-DNA-binding protein 1 (ZBP1) could bind dsDNA directly, and activate the innate response via activating IRF3 and NFκB.^72^ In addition, it can sense RNA to trigger necroptosis via recruiting RIPK3 to phosphorylate and activate MLKL, which leads to virus-infected cell death.^73^ LRRFIP1 was reported to recognize both RNA and DNA from viruses or bacteria, which could lead to accumulation and subsequent translocation into the nucleus of β-catenin to enhance the transactivation of IRF3.^74^ DEAD-box helicase 41 (DDX41) could sense dsDNA or c-di-GMP to induce innate immunity through binding and activating STING directly to activate the transcription factors IRF3 and NFκB.^75^ Protein kinase RNA-activated (PKR) can recognize long viral dsRNA during HCV infection, and then undergoes dimerization and autophosphorylation. Phosphorylated PKR can block translation initiation via phosphorylating eIF2 on Ser51 to induce cell death,^76^ and activate NFκB via phosphorylating IKKB to induce innate immune.^77^ Another group of non-RLR RNA helicases could also sense RNA and mediate inflammasome, or enhance RLR signaling to enhance IFN response and virus infection inhibition effect.^78^ One study reported that, upon binding to dsDNA, the NOD-like receptor family CARD domain containing 3 (NLRC3) could unleash STING from its sequestration, which induces STING mediated immunity activation.^79^

## The combination of nuclei acid-sensing immunity with ICIs as a promising strategy in cancer immunotherapy

Given the primary role of nuclei acid-sensing induced immune responses in activating T cell functions, there are various studies pointing out the advantages of activating the nuclei acid-sensing immunity to enhance the effect of ICIs in cancer immunotherapy (summarized in Table 1).

**Table 1:** Nucleic acid sensor agonists in combination with immune checkpoint inhibitors (ICIs) in mouse models

Agents	targets	Cancer types	Biological roles	Reference(PMID)
5′- Triphosphate RNA	RIG-I	Acute myeloid leukemia Colon cancer Melanoma	• Induces type I IFNs expression • Increases the infiltration of CD4^+^ and CD8^+^ T cells • Induces programmed death ligand 1 (PD-L1) expression on AML cells • Establishes therapeutic sensitivity to immune checkpoint inhibitors	31740809 30379158 30852164
Poly (I:C)	MDA5	Pancreatic cancer	• Induces the expression of type I IFNs and other proinflammatory cytokines in tumor tissue • Activates DCs, induces Th1 polarization, upregulates the expression of Fas and MHC-I, induce Fas-mediated apoptosis and cytotoxic T lymphocyte-mediated lysis	25012502
ARNAX	TLR3	Lymphoma	• Induces Type I IFNs production • Promotes accumulation of CD8^+^ T cells and CD8α^+^ DCs into tumors • Synergistically induces anti-tumor immunity with the PD-L1 antibody.	28564605
BO-112	TLR3/MDA5	Melanoma, Colon carcinoma Breast carcinoma	• Induces the production of type I IFNs, IFNγ • Activates of CD8^+^ T lymphocytes and tumor antigen-specific cytotoxic T lymphocytes • Induces tumor regression	31046839
Resiquimod	TLR7/8	Pancreatic cancer	• Induces CD8+ T cell proliferation and effector function, • Decreases Th2 polarization among CD4+ T cells	31615993
Imiquimod	TLR7	Breast cancer	• Activates NK cells, macrophages and B lymphocytes in combination with laser irradiation can. • Induces CD8^+^, CD3^+^, CD4^+^ and PD-1^+^ T cells infiltration of distant tumors. • Increases the response to anti-PD-1 antibody in combination with irradiation	30339018
3M-025	TLR7/8	Melanoma	• Increases the level of CCL2 chemokines and infiltration of M1 phenotype-shifted macrophages • Induces the production of type I IFN, IFNγ • Activates CD8^+^ T cells, B cells, and pDC to induce tumor suppression • Potentiates checkpoint blockade therapy	25252955
1V270	TLR7	Head and neck squamous cell carcinoma	• Increases the ratio of M1 to M2 tumor-associated macrophages • Promotes the infiltration of tumor specific IFNγ producing CD8^+^ T cells • Enhances the efficacy of anti-PD-1 treatment	28931759
SD-101	TLR9	Colon carcinoma	• Stimulates the TLR9 of pDCs to release IFNs and mature • Induces the infiltration and expansion of CD8^+^ T cells. • Overcomes resistance to PD-1 blockade	27799536
CMP-001	TLR9	Colon carcinoma Pancreatic cancer	• Increases the production of IFNγ, IL-6, and IL-12 Induces the infiltration of CD8+ T and NK cells • Elicits anti-tumor immune response and improves the survival	32409965
STINGVAX	STING	Melanoma	• Increases the tumor-infiltrating CD8^+^ IFNγ^+^ T cells Induces the expression of PD-L1 in tumor • Overcomes tumors resistant to PD-1 blockade	25877890
ADU-S100	STING	HPV^+^ oral cancer	• Synergistically induces tumor regression in combination with PD-LA antibody and CTLA-4 antibody	31533840
2′3′-c-di-AM (PS) 2 (Rp, Rp)	STING	High-grade serous ovarian cancer	• Induces the production of IFNs • Increases antigen presentation and MHC genes in tumors • Increases the tumor infiltration of PD-1^+^, CD69^+^ CD62L^−^, CD8^+^ T cells • Synergistically induces tumor regression in combination with PD-LA antibody	30046165
Cyclic di-GMP	STING	Prostate cancer	• Increases T cell infiltration and reduces suppressive myeloid polarization • Potentiates systemic checkpoint modulation	28674082

### The usage of sensor agonists

#### RIG-I like receptor ligands

RIG-I like receptor ligands have been proved promising for the treatment of malignancies such as breast cancer, melanoma, pancreatic cancer and acute myeloid leukemia (AML) in preclinical models.^31,80–82^ RIG-I activation via short 5′ppp-RNA induced tumor rejection, and immunological memory formation in acute myeloid leukemia (AML) model as a result of type I IFN induction to increase the level of CXCL10 to activate CD4^+^ and CD8^+^ T cells.^82^ Accordingly, 5′ppp-RNA treatment could sensitive ICIs treatment in vivo.^82–84^ In primary human melanoma tissues, high expression of RIG-I was associated with T cell receptor and antigen presentation pathway activation, the prolonged overall survival of patients, and durable clinical responses to anti-CTLA-4 checkpoint blockade.^85^ Thus, the high expression of RIG-I in cancer cells may indicate a better response to immune checkpoint blockade which still needs further research.

MDA5 activation via poly I:C in pancreatic cancer cells, induced apoptosis and the production of type I IFN and pro-inflammatory cytokines, which led to the activation of DCs. And the activated CD8α^+^ DCs engulfed apoptotic tumor material and cross presented tumor-associated antigen to naïve CD8^+^ T cells, which induced cytotoxic T lymphocyte (CTL) mediated lysis.^86^

Accordingly, phaseI/II clinical study was performed to evaluate the safety, tolerability, and anti-tumor activity of intratumoral (IT)/intralesional (IL) injections of the agonist of RIG-I, MK-4621 (RGT100), as monotherapy or in combination with pembrolizumab in participants with advanced solid tumors (NCT03065023 and NCT03739138, summarized in Table 2).

**Table 2:** The clinical trials of nucleic acid sensor agonists in combination with immune checkpoint inhibitors (ICIs)

Target	Agent	Indication	Combination	Clinical trial ID	phase
RIG-I	MK-4621/JetPEI™	Solid tumors	Pembrolizumab	NCT03739138	I
TLR3	Poly(I:C12U) (Rintatolimod; Ampligen)	Breast Cancer	Pembrolizumab	NCT03599453,	I
		Ovarian Cancer		NCT03734692	I,II
		Colorectal Adenocarcinoma		NCT04119830	II
		Melanoma		NCT04093323	II
	Poly-ICLC (Hiltonol)	Colon Cancer	Pembrolizumab	NCT02834052	I,II
		Solid tumors	Nivolumab Pembrolizumab Atezolizumab Durvalumab	NCT03721679	I,II
		Ovarian Cancer	Nivolumab	NCT04024878	I
		Solid tumors	Nivolumab Pembrolizumab	NCT03633110	I,II
		Melanoma	Nivolumab Ipilimumab	NCT03929029	I
				NCT03597282	I
		Lung cancer	Pembrolizumab	NCT03380871	I
		Prostate Cancer	Nivolumab	NCT03835533	I
		Breast Cancer	Pembrolizumab	NCT03362060	I
		Follicular Lymphoma	Nivolumab	NCT03121677	I
		Kidney Cancer	Ipilimumab	NCT02950766	I
		Hepatocellular Carcinoma	Nivolumab Ipilimumab	NCT04248569	I
		Glioma	Nivolumab	NCT02960230	I
		Colorectal Cancer Pancreatic Cancer	Nivolumab Ipilimumab	NCT04117087	I
		Breast Cancer	Durvalumab	NCT02826434	I
TLR3/ MDA5	BO-112	Colorectal Cancer Gastric Cancer Oesophageal Cancer	Pembrolizumab	NCT04508140	II
		Sarcoma	Nivolumab	NCT04420975	NA
TLR7	Imiquimod (R-837)	Melanoma	Pembrolizumab Toripalimab	NCT03276832, NCT04072900	I
		Solid Tumors	Anti-PD-1 antibody	NCT04116320	I
		Breast Cancer	Pembrolizumab	NCT03982004	I
TLR7/8 and RIG-I	CV8102	Solid Tumors	Anti-PD-1 antibody	NCT03291002	I
TLR8	Motolimod (VTX2337)	Head and Neck Squamous Cell Carcinoma	Nivolumab	NCT04272333	I
		Ovarian Cancer	Durvalumab	NCT02431559	I,II
		Head and Neck Cancer	Nivolumab	NCT03906526	I
TLR9	Tilsotolimod (IMO-2125)	Solid Tumor	Nivolumab; Ipilimumab	NCT03865082	II
		Advanced Cancer	Nivolumab; Ipilimumab	NCT04270864	I
		Melanoma	Ipilimumab	NCT03445533	III
	Lefitolimod (MGN1703)	Advanced Cancers	Ipilimumab	NCT02668770	I
	SD-101	Pancreatic Adenocarcinoma	Nivolumab	NCT04050085	I
		Prostate Cancer	Pembrolizumab	NCT03007732	II
		Breast Cancer	Pembrolizumab	NCT01042379	NA
	CMP-001	Melanoma	Pembrolizumab	NCT03084640, NCT02680184	I
			Nivolumab	NCT04401995	II
		Melanoma Lymph Node Cancer	Nivolumab	NCT03618641	II
		Lymphoma	Pembrolizumab	NCT03983668	I,II
		Colorectal Cancer	Nivolumab, Ipilimumab	NCT03507699	I
		Advanced Cancer	Avelumab	NCT02554812	II
	IMO-2125 (Tilsotolimod)	Melanoma	Ipilimumab	NCT03445533	III
		Solid Cancer	Nivolumab Ipilimumab	NCT03865082	II
STING	MIW815(ADU-S100)	Head and Neck Cancer	Pembrolizumab	NCT03937141	II
		Solid Tumors Lymphomas	Ipilimumab	NCT02675439	I

#### TLRs agonists

Given the critical roles of TLRs in triggering innate immunity, various agents have been found to modulate immunity to anti-tumors.^87,88^

An artificial ligand to TLR3, a synthetic DNA-dsRNA hybrid molecule (ARNAX) can activate antigen-presenting dendritic cells to induce cytotoxic T lymphocyte proliferation through TLR3-TICAM-1-IRF3-IFNβ pathway.^89^ On the other hand, it can establish a tumor suppression microenvironment through inducing the expression of DCs recruitment-related genes including CCL4, CCL5, and CCL27. In addition, treatment of ARNAX with tumor-ssociated antigen could induce mRNA expression of genes related to cytotoxicity (IFNγ, Gzmb, and Prf1) and chemokines recruiting the CTLs (Cxcl9 and Cxcl10) in tumors, induce effector CTLs infiltration, and facilitate Th1-type anti-tumor immunity in mouse model. Nevertheless, the combination of the anti-PD-L1 antibody with TLR3 specific adjuvant (ARNAX + tumor Antigen) induced a more effective tumor regression response in a mouse model.^90^ And the evaluated proliferation of antigen-specific CTLs of human peripheral blood mononuclear cells (PBMCs) was observed induced via ARNAX treatment combined with TAA. However, ARNAX has not yet entered clinical development.

Poly-ICLC (Hiltonol) is a synthetic Poly-IC that can induce tumor specific NK cells, CTLs, and NK-T cell-mediated immune responses via activating TLR3 and MDA5.^91,92^ In addition, poly-ICLC was shown to be an effective adjuvant to prime antigen specific CD8^+^ T cells and prolong survival In murine models of glioma and melanoma.^93^ And various clinical studies have revealed the contributions of Poly-ICLC alongside tumor-specific antigens to improve immune responses.^91,92,94^

BO-112 is a nanoplexed form of Poly I:C, which may activate sensors such as TLR3 and MDA5. Intratumoral BO-112 treatment leads to remarkable tumor regression dependent on type I IFN and gamma-interferon in the mouse models.^89^ Furthermore, more abundant CD8^+^ T lymphocytes and tumor antigen specific cytotoxic T lymphocytes were found following intratumoral BO-112 treatment. And intratumoral BO-112 administration showed unilaterally to bilaterally efficacy in tumor bearing mice conjunction with systemic anti-PD-L1 monoclonal antibodies.^89^

The agonist of TLR7, Imiquimod (also known as R-837) is an immune response modifier and has been approved by U.S. Food and Drug Administration (FDA) for treatment of superficial basal cell carcinoma, genital warts and actinic keratosis.^95^ In the breast cancer model, Imiquimod in combination with laser irradiation can activate NK cells, macrophages and B lymphocytes via TLR-7 induced cytokines (IFN-α, IL-6 and TNF-α) to further enhance the activation of immune response. And it can induce much more CD8^+^, CD3^+^, CD4^+^ and PD-1^+^ T cells in distant tumors. Thus, Imiquimod in combination with irradiation can increase the response to anti-PD-1 antibody.^96^ Moreover, it was described that several cases of melanoma patients successfully treated with combination of ipilimumab and Imiquimod.^97,98^

The TLR7/8 agonist Resiquimod (R848) was revealed to activate pDCs and cDCs in vivo, which enhanced T cells priming in regional lymph nodes. And combining PD-L1 antibody treatment with resiquimod is useful to reduce tumor growth in two PD-L1 blockade-resistant tumor models.^99^ However, several clinical studies revealed that resiquimod as adjuvants for a melanoma vaccine is not sufficient to induce consistent antigen- specific CD8^+^ T-cell responses.^100,101^ 3M-025 is another TLR7/8 agonist, which could induce an increased level of CCL2 chemokines, infiltration of M1 phenotype-shifted macrophages, cytotoxic T cells in melanoma mouse model.^50^ Meanwhile, TLR7/8-agonist-loaded nanoparticles with tumor associated macrophage (TAM) avidity could reverse the polarization of TAM from the M2-like phenotype (anti-inflammatory) to M1-like phenotype (pro-inflammatory) and inhibit tumor growth in a CD8^+^ T cell-dependent manner in vivo.^102^ Accordingly, the combination of TLR7/8 with anti-PD-1 and anti-CTLA-4 resulted in a synergy of tumor regression.^50^

Motolimod (VTX-2337) is a specific TLR8 agonist that activates NK cells and augments antibody-dependent cellular cytotoxicity (ADCC).^103^ In addition, motolimod can facilitate the activation of NK cells and dendritic cell cross-priming of tumor specific CD8^+^ T cells in head and neck cancer.^104^

IMO-2125, the agonist of TLR9, was revealed to lead to the production of Th1-type cytokines and chemokines.^105^ Treatment of IMO-2125 directly into one tumor leads to potent tumor regression of the injected and uninjected distant tumors via the CD8^+^ T cell directed Th1 response with a long-term tumor specific memory in the colon carcinoma mouse model.^106^ And phase I/II clinical trials have shown that the combination of IMO-2125 and Ipilimumab is well-tolerated and actively promotes anti-tumor immune responses in melanoma. The treatment induced responses even in distant tumors that did not receive IMO-2125 treatment (NCT02644967).^107^

Intratumoral treatment of TLR9 agonist, SD-101, which stimulates the TLR9 of pDCs, can induce pDCs to release IFNs and mature, thus could induce the infiltration and expansion of CD8^+^ T cells. And accordingly, SD-101 treatment could overcome resistance to PD-1 blockade in the colon carcinoma mouse model.^108^ CMP-001, another agonist of TLR9 can also facilitate the anti-tumor immune response including the increased expression of chemokines, pro-inflammatory cytokines, and the infiltration of CD8^+^ T and NK cells, which improves the survival in colon carcinoma and pancreatic cancer.^109^ Furthermore, It’s inspiring that the agonists of TLR9, CMP-001, and SD-101, could stimulate TLR9 and induce pDCs to secrete type I IFNs and inflammatory chemokines, and facilitate cytotoxic T cells infiltration to potentially overcome the resistance of PD-1 blockade in metastatic melanoma patients.^110–112^

Based on these promise evidence in the preclinical studies, several clinical trials were conducted with TLR agonists in combination with ICIs to evaluate the potential clinical efficacy (summarized in Table 2).

#### STING agonists

Since STING is widely expressed in various types of cells in tumor tissues, synthetic STING agonists such as cyclic dinucleotide (CDNs) or cyclic di-GMP (CDG), could stimulate STING pathway in DCs, macrophages, B cells and other leukocytes to induce the production of Type I IFNs. The IFNs, in an autocrine or paracrine manner, could facilitate the antigen presentation to CD4^+^ and CD8^+^ T cells of DCs, and the priming of NK cells, which potentiates antitumor responses.^113^ Many efforts are focused on the development of modified CDNs or other small molecules.^114^ STING agonists have shown significant therapeutic benefit via increasing DCs mediated CD8+ TILs infiltration into the tumor microenvironment in melanoma, acute myeloid leukemia, breast and colorectal cancer mouse models.^115–117^ STING agonist formulated cancer vaccine (STINGVAX) treatment could increase the tumor infiltrating CD8^+^ IFNγ^+^ T cells with marked PD-L1 up-regulation in the melanoma mouse model, which could sensitize the PD-1 blockade.^115^ STING agonists induced systemic pro-inflammatory cytokines, antigen processing and presentation in DCs as well as elevated PD-1^+^ CD8^+^ T cell ratio in mice with high-grade serous ovarian carcinoma. The combination of the carboplatin with STING agonist and PD-1 blockade significantly prolonged the survival of these mice.^118^ Similarly, STING agonists such as ADU-S100 (also known as MIW815 or ML RR-S2 CDA), 2′3′-c-di-AM (PS) 2 (Rp, Rp), and cyclic di-GMP could enhance the therapeutic efficacy of anti-PD-1/PD-L1 immunotherapy in colon cancer, HPV^+^ oral cancer, pancreatic cancer, prostate cancer and so on.^119–121^

Therefore, the combination of STING agonists with ICIs is promising to enhance the effect of ICIs in antitumor therapy by activating innate and adaptive immunity. As a result, there are several clinical trials being performed (summarized in Table2).

### Induction of cytoplasmic RNA

In addition, there are other strategies to stimulate nuclei acid sensors such as induction of cytoplasmic RNA or DNA from the host to mimic the microbial infections. For example, the inhibition of histone H3K4 demethylase LSD1 in tumor cells increases the endogenous retroviral sequences (ERVs) transcripts, which allows the formation of dsRNA. And LSD1 could induce the protein stability of AGO2 through enhancing the methylation of AGO2. AGO2 is the key component of the RNA-induced silencing complex (RISC) complex, which mediates the RNA silence. Therefore, inhibition of LSD1 increases the accumulation of dsRNA, which is sensed by MDA5 and TLR3, and then activates the expression of IFNs and MHC-I expression in tumor cells. In addition, a synergy in controlling tumor growth between LSD1 inhibition and PD-1 blockade was observed in the B16 tumor and triple-negative breast cancer (TNBC) tumor mouse models as a result of the enhanced tumor immunogenicity, T cell infiltration, and anti-tumor T cell immunity.^122,123^ Furthermore, LSD1 is overexpressed in tumors compared with normal tissues in a broad of cancers, which indicates LSD1 is a promising target to enhance the effect of ICIs.^122^

Besides, the treatment of DNA-demethylating agent 5-AZA-CdR in tumor cells can induce the transcription of specific ERVs, and increase cytoplasmic dsRNAs. And the increase of dsRNAs is detected via MDA5, which further induces IFN response to anti-tumor.^124,125^ In addition, 5-AZA-CdR treatment can potentiate the anti-tumor effect of anti-CTLA-4 antibody in the breast cancer mouse model, which may be associated with high viral defense.^126^

Recent studies showed that gain-of-function mutations of MDA5 (GOF MDA5) lead to aberrant activation of its signaling, resulting in a variety of immune disorders, such as Aicardi-Goutières syndrome (AGS) and systemic lupus erythematosus (SLE).^127–129^ Further study indicates that the Alu: Alu hybrids formed of inverted repeats (IRs) which are abundant in the cytosol, are the primary ligands for GOF MDA5, while wild type MDA5 has limited ability to recognize Alu: Alu hybrids. Interestingly, under the deficiency of ADAR1 (an A to I RNA editor), the unmodified Alu: Alu hybrids could activate wild type MDA5 significantly,^130^ which induces the production of Type I IFNs significantly. On the other hand, upon IFNs response, ADAR1 deletion could activate another dsRNA sensor, PKR to induce apoptosis in several cancer cell lines.^131^ Furthermore, loss of ADAR1 can overcome the resistance to PD-1 checkpoint blockade as a result of a significant increase in IFNs production and immune cells, including CD4^+^ T cells, CD8^+^ T cells, NK cells, and decreasing in M2 type myeloid cells in mouse model.^132^

Cyclin-dependent kinases 4 and 6 (CDK4/6) inhibitor have shown significant activity on inhibiting the phosphorylation retinoblastoma (RB) tumor suppressor, and subsequently inducing G1 cell cycle arrest in tumor cells.^133^ Apart from inducing cell cycle arrest, CDK4/6 inhibitor could reduce the activity of DNA methyltransferase 1 (DNMT1) in breast cancer cells, which reduces DNA methylation at ERV sequences and increases the levels of dsRNA within tumor cells. High levels of dsRNA trigger RNA recognition receptors such as RIG-I, LGP2 and MDA5, which in turn stimulates the production of type I IFNs and hence enhances tumor antigen presentation. In addition, CDK4/6 inhibitors suppress the proliferation of Tregs and promote cytotoxic T cell- mediated clearance of tumor cells.^134^ In vitro and in vivo studies revealed that CDK4/6 inhibition resulted in increased anti-tumor activity and sensitivity to the PD-1 blockade.^135^

Therefore, the above studies indicate that induction of cytoplasmic RNA to induce innate immune in tumors via deleting LSD1 or ADAR1, or treatment of DNA-demethylating agent or CDK4/6 inhibitors, showed a promising effect to combine with ICIs to anti-tumor. However, further studies probably will focus on clinical translation such as LSD1 and ADAR1 inhibitor invention.

### Induction of cytoplasmic DNA

Since several pivotal research revealing surveillance of micronuclei by cGAS, much attention was paid to the synergy between DNA damage and ICIs.^136,137^ Accumulation of cytoplasmic DNA in micronuclei of tumor cells, which was induced by radiation or chemotherapy agents (such as cisplatin and etoposide), DNA damage repair blockage (such as PARP inhibition and BRCA1 deficient), and CHK1 inhibition to prevent cell cycle arrest during DNA damage repair, could activate cGAS-STING pathway to induce the innate immunity.^138–141^

However, there are different mechanisms underlying. For instance, radiation therapy could sensitize the ICIs, which might be mediated via resident and infiltrating polyclonal T cells, diversity of T cell receptor(TCR) repertoire of intratumoral T cells after radiation.^142,143^ Ataxia Telangiectasia Mutated (ATM) is an important component in DNA damage response (DDR) and plays a critical role in responding to radiation treatment induced DNA double strand breaks. In pancreatic cancer, the inhibition of ATM alone, or in combination with radiation, could increase the phosphorylation of the tyrosine kinase Src and subsequent phosphorylation of TBK1, which will increase the transcription of type I IFNs and the activation of innate immunity. As a result, ATM inhibition sensitizes cancer cells to ICIs.^144^

CHK1 inhibitors or PARP inhibitors induced STING-dependent CXCL10 transcription from small cell lung cancer cell lines *in vitro*. Since CXCL10 is a chemokine important for recruiting immune cells, its increased production would sensitize cancer cells to ICIs treatment. Indeed, in the small cell lung cancer mouse model, PARP or CHK1 inhibition significantly potentiated the anti-tumor effect of PD-L1 blockade via STING mediated IRF3 activation, thereby inducing type I IFNs production and innate immune activation.^139^

BRCA1 and BRCA2 are key components in DNA damage repair through homologous recombination (HR).^145^ BRCA1 deficiency-induced DNA damage could increase the micronuclei release into the cytoplasm of breast cancer cells. The micronuclei are sensed by cGAS/STING and induce CXCL10 and CXCL5 production, which increase the CD4^+^ and CD8^+^ T cells infiltration. And the expression of PD-L1 in tumor cells is upregulated dependent on STING activation.^146^ And HR deficiency and inhibition of PARP have been shown to produce synthetic lethality. PARP inhibition in BRCA1-deficient TNBC mouse model, could facilitate CD8^+^ T cell infiltration and antitumor efficacy through activating cGAS/STING pathway in tumor cells thereby paracrine activation of dendritic cells.^147,148^ In addition, the combination of PARP1/2 inhibitor and anti-PD-1 antibody demonstrated synergistic antitumor activities in BRCA1-deficient TNBC mouse model.^147^ Besides, in BRCA1-deficient ovarian cancer cells, treatment of PARP inhibitor combined with another immune checkpoint inhibitor, a CTLA-4 antibody, could increase T cell recruitment and activation, cytokine production, and lasting systemic effector/memory T cell immunity, which eventually bring about the treatment synergy.^149^

Furthermore, a study revealed that the defects in double strand break (DSB) repair protein such as Ku70/80 complex or BRCA2 lead to ATR/CHK1 activation and subsequent increasing STAT1/STAT3 phosphorylation and IRF1 expression, which induces PD-L1 transcription.^150^ Besides, ATM and CHK1 inhibition could increase the IFNs autocrine and paracrine, which increase the STAT1/STAT3 phosphorylation via IFN receptors. And the phosphorylation of STAT1/STAT3 increases IRF1 expression, which induces PD-L1 expression on tumor cells. And the upregulation of PD-L1 expression could provide a theoretical basis for combination therapy with ICIs in another way.^144,150,151^

### Oncolytic viruses (OVs)

During the past decades, oncolytic viruses (OVs) have been generated from both DNA viruses and RNA viruses.^152,153^ There are numerous advantages of OVs as cancer therapeutic agents. OVs directly and selectively kill tumor cells through immunogenic cell death. In addition, viral elements (such as DNA and RNA) can bind to nucleic-acid receptors (such as TLRs, cGAS-STING, MDA5, RIG-I, and so on), which triggers the release of type I IFNs and chemokines.^154–156^ Moreover, the death of tumor cells results in the release of soluble tumor-associated antigens, viral pathogen-associated molecular patterns (PAMPs), and cell derived damage-associated molecular patterns (DAMPs). PAMPs and DAMPs could recruit and activate APCs to engulf soluble tumor antigens and migrate to regional lymph nodes to prime adaptive T cell response against tumor. On the other hand, virus infection can induce the expression of MHC class I, and recruitment of tumor-specific CD8^+^ T cells.^157^ Furthermore, excessive interferon production may induce immune-suppressive environment through increasing the expression of checkpoint molecules, such as PD1, PD-L1, and CTLA-4.^155,158,159^

Early clinical trials of OVs have shown its safety and promising responses in a variety of tumors.^160^ And accordingly, it’s promising to combine the OVs with ICIs to accelerate the anti-tumor immune response and remove the barriers of T cell-mediated tumor killing. Indeed, preclinical experiments revealed that OVs could increase the sensitivity of tumors to ICIs via activating T cell activity.^161–164^ In line with these preclinical studies, there are multiple clinical trials adopted to investigate the combination of oncolytic virotherapy with ICIs in cancer therapy (summarized in Table 3).^164^ For example, one phase Ib clinical trial showed that oncolytic virotherapy could improve the efficacy of anti-PD-1 therapy with a higher overall response rate and complete response in metastatic melanoma via enhancing the CD8^+^ infiltration, PD-L1 expression, as well as IFN-γ expression.^165^ And an ongoing Phase III clinical trial is currently underway to better evaluate the efficacy of this combination therapy in patients with stage IIIB-IV melanoma (NCT02263508). Therefore, oncolytic virotherapy showed attractive interests in cancer therapy, especially the in combination of ICIs.

**Table 3:** The clinical trials of the oncolytic virus in combination with immune checkpoint inhibitors (ICIs)

Oncolytic virus	Agents	Indication	Combination	Clinical trial ID	phase
Vaccinia Virus	Pexa-Vec	Renal Cell Carcinoma	REGN2810 (Anti-PD-1)	NCT03294083	I
	Pexa-Vec	Solid Tumor	Ipilimumab	NCT02977156	I
	ChAdOx1-MVA 5T4 vaccine	Prostate Cancer	Nivolumab	NCT03815942	I,II
	p53MVA Vaccine	Solid tumor	Pembrolizumab	NCT02432963	I
	Pexa-Vec	Colorectal Cancer	Tremelimumab, Durvalumab	NCT03206073	I,II
	p53MVA Vaccine	Ovarian Cancer	Pembrolizumab	NCT03113487	II
	PROSTVAC	Prostate Cancer	Nivolumab, Ipilimumab	NCT03532217	I
	p53MVA Vaccine	Solid tumor	Pembrolizumab	NCT02432963	I
	TG4010	Non-Small Cell Lung Cancer	Nivolumab	NCT02823990, NCT03353675	II
Herpes simplex virus	OH2	Solid Tumor Melanoma	Keytruda	NCT04386967	I,II
	ONCR-177	Solid Tumor	Pembrolizumab	NCT04348916	I
	OrienX010	Melanoma	JS001	NCT04206358	I
	OH2	Solid Tumor	HX008	NCT03866525	I,II
	OrienX010	Melanoma	Treprizumab	NCT04197882	I
	RP1	Solid Tumor	Nivolumab	NCT03767348	I,II
	ADV/HSV-tk	Non-small Cell Lung Cancer Triple-negative Breast Cancer	Pembrolizumab	NCT03004183	II
	RP2	Solid Tumor	Nivolumab	NCT04336241	II
	Talimogene Laherparepvec	Melanoma	Pembrolizumab	NCT02965716	II
	HF10	Melanoma	Nivolumab	NCT03259425	I,II
	Talimogene laherparepvec	Hepatocellular carcinoma	Pembrolizumab	NCT02509507	I
	Talimogene Laherparepvec	Sarcoma	Pembrolizumab	NCT03069378	II
	Talimogene Laherparepvec	squamous cell carcinoma of the head and neck	Pembrolizumab	NCT02626000	II
	Talimogene Laherparepvec	Melanoma	Pembrolizumab	NCT02263508	III
	Talimogene Laherparepvec	Breast Cancer	Ipilimumab Nivolumab	NCT04185311	I
Adenovirus	ONCOS-102	Melanoma	Pembrolizumab	NCT03003676	I
	DNX-2401	Brain Cancer	Pembrolizumab	NCT02798406	II
	VCN-01	Head and Neck Neoplasms	Durvalumab	NCT03799744	I
	Ad-MAGEA3	Non-Small Cell Lung Cancer	Pembrolizumab	NCT02879760	I,II
	ONCOS-102	Colorectal Cancer	Durvalumab	NCT02963831	I,II
	Enadenotucirev	Colorectal Cancer	Nivolumab	NCT02636036	I
	OBP-301	Solid tumor	Pembrolizumab	NCT03172819	I
	ONCOS-102	Melanoma	Pembrolizumab	NCT03003676	I
Measles virus	TMV-018	Gastrointestinal Cancer	Anti-PD-1 checkpoint inhibitor	NCT04195373	I
Reovirus	Pelareorep	Breast Cancer	Retifanlimab	NCT04445844	II
Coxsackievirus	CVA21	Non-Small Cell Lung Cancer	Pembrolizumab	NCT02824965	I

## Conclusions

**Fig. 5:**
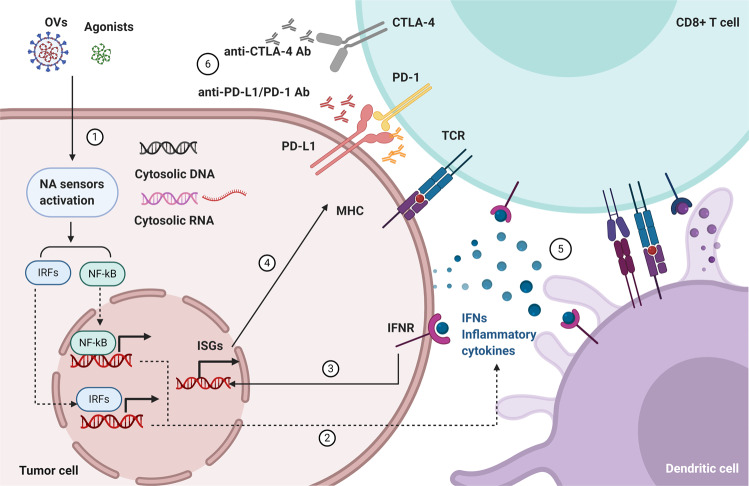
Targeting nucleic acid sensing immunity to sensitize immune checkpoint inhibitors (ICIs) in cancer therapy. The working model of targeting nucleic acid-sensing immunity to sensitize ICIs in cancer therapy. 1. The induction of cytoplasmic DNA, RNA, the agonists of nucleic acid sensors, and oncolytic viruses (OVs) can stimulate the nuclei acid sensors. 2. Activated sensors mediate the production and secretion of IFNs and cytokines in tumor cells and DCs. 3. The secreted type I IFNs will act on producing and neighboring cells via IFNRs. 4. Type I IFNs induce the expression of PD-L1 and MHC-I in cancer cells. 5. The production of IFNs can promote the maturation of DCs to improve cross-priming with T cells, and activate NK cells to kill targeted tumor cells. 6. Unleashing the nucleic-acid-sensing mediated innate immunity fuels the accelerators of T cells, and ICIs release the multiple brakes on T cells, which can induce the elimination of the tumor cells

Nucleic acid-sensing pathway plays critical roles in activating T cells functions in pathogen infection as well as cancer. Generally, high expressions or gain-of-function mutations of nucleic acid sensors, treatments with extensive ligands or agonists of the receptors in tumors, or induction of nucleic acid can mimic the pathogen infections to activate nucleic acid-sensing signalings. And the stimulation of nucleic acid-sensing signalings induces the transcription of type I IFNs and other cytokines, which are important for remodeling tumor microenvironment. As a result, more immune cells will be recruited and more cancer cells are induced to apoptosis. More importantly, PD-L1 expression in cancer cells is increased, indicating that ICIs would be more effective. Thus, activating nucleic acid-sensing innate immunity seems a rational strategy in synergy with ICIs to anti-tumors (Fig. 5).

However, when considering the combination of sensor agonists, the expression and the relevance of sensors can be various in different cancers. For instance, the high expression of RIG-I in ovarian cancer is correlated with higher tumor grade and poor outcome, as well as the higher expression of PD-L1 and the regulatory T cell-specific transcription factor FoxP3.^166^ In contrast, the low expression of RIG-I in hepatocellular carcinoma patients is positively correlated to short survival.^167^ Thus, the expression and the relevance of sensors in different tumor types should be considered in the combination of these sensor agonists with ICIs.

Emergence evidence revealed that nucleic acid receptors sense cytoplasmic RNA or DNA produced by chemotherapy, radiation therapy or DNA damage. For instance, AIM2 could sense ionizing radiation-induced DNA damage in the nucleus, to trigger caspase-1 mediated cell death in intestinal epithelial cells and bone marrow cells.^168^ However, deficiency of AIM2 could increase the phosphorylation of AKT at Ser473 to increase the proliferation of mice colon cancer cells independent of AIM2 mediated inflammation.^169^ cGAS could recognize cytoplasmic DNA or micronuclei produced by chemotherapy, radiation therapy, or DNA damage to activate innate immune.^136,137^ Nevertheless, DNA damage could induce nuclear translocation of cGAS, which could sense DNA double stranded breaks, and recruits PARP1 to inhibit DNA homologous recombination (HR) and promotes tumorigenesis in certain types of cancer.^170^ TLR3 signaling may also participate in pro-inflammatory reactions contributing to tumorigenesis.^171^ Taken together, when considering the strategies of activating nucleic acid-mediated immunity to sensitive the ICIs, researchers should keep in mind that other signaling pathways that could lead to either pro-tumor or anti-tumor effects need to be carefully considered. Interestingly, inhibition of ATR destabilizes PD-L1, which could sensitize tumor cells to T cell-mediated killing.^172^ Therefore, the effect of the combination of ATR inhibition and ICIs in cancer immunotherapy needs further study.

Nevertheless, unleashing the nucleic-acid-sensing-mediated innate immunity fuels the accelerators of T cells, and ICIs release the multiple brakes on T cells. Accordingly, it’s promising to combine harnessing the nucleic acid-mediated immune response with ICIs in cancer immunotherapy. However, further researches should pay more attention to the homeostasis of the tumor immune-microenvironment, and emergent clinical translational studies are still needed for this synergy strategy.
